# Supplementary material for: Chromothripsis during telomere crisis is independent of NHEJ, and consistent with a replicative origin
Source: Genome Res. 2019 May;29(5):737–49. doi: 10.1101/gr.240705.118 (PMC6499312; doi:10.1101/gr.240705.118)
Supplement: Supplemental Material [file supp_gr.240705.118_Supplemental_file_1.zip › contigs/annotated_contigs/DB45/contig.2.DB45_length_940_mean_cov_4.58085106383.docx]

**DB45_length_940_mean_cov_4.58085106383**

```
CCAGGCCGGGCTGAGAACTCCCTATAGCAGTAAATCCTGCTGGTGCTCGAGTTGCCTCAAGGAAAGGGTTACTGCCTGGCTTCCCATGG
 >chr22:39823458-39823825 - E=8e-206
ATGCTTTAATTCAGAGGATAAGTTTGCTCCCAGTTGAGGCTGACCCAATAAAAATATGGTTGCAATAAAAACAGGCCAGAGAAACTCCA

ATAACCCTGGTGTCCATGGGAGAACAAATCTGTCTGTGGATGCCTGAGCTTTATCTAAGATTTGCTTGTTTAAAGCAAAGAAAAAGAAA

TGCAGCCCTCAGGAACCGAAGGTCTTAGTGCTGAGCAGCACTGGGGGGAGCACGGGAAAGAGCAGCCACCTCTTTTCCTGAGAACTTGG

TTGAAGAATGA|AG|CTGAAAACTGTGCTTCAGAAAGAAGGAAACTGAATCTACACAAAAGAAATGAAATACAAGGAACAACAGTGAGC
              >chr11:94215636-94216207 + E=0e+00
AAAGAAATAGGTAAACATGTATATAGGGTGGGGAAACAGTTTTCAAAACAGAATGCAATTTAAAAATAAGATGAAAGAAAGAAAATACT

GAAGTTAAAAAATGTCCCGGCTGGGTGTGGTGGCTCACGCCTATAATCTCAACACTTTCGGAGGCCAAGTCGGGAGGATCACTTGAGGT

CAGGAGTTCAAGACCAGCCTCACCAACCTGGTGAAACCCCGTCTCTACTAAAAACGCATAAAAAAATTAGCTGGGCATGGTGGTGCACA

CTTGTAGTCCCAGCTACTCAGGAGGCTGAGGCAGAAGAACTGCTTGAACCTGGGAGGTGGAGGTTGCAGTGAGCCAAGATCGTGCCTCT

GCACTCCAGCTTGGGTGACAGTAAGACTCCTTCTCAAAAATAAAAAATAAAAAATAAAAAATGTGCCAAGTTTTAGATTTGAATAGAAG

GAGGGTAAAGACATTGATCACAATTTTGAAAAACAAGGACAAAGTATTATAC
```
